# Microwave Processing at the 915 MHz Frequency for Efficient Cleavage of Cellulose and Lipids in Vegetable and Meat Wastewater Treatment

**DOI:** 10.1111/1750-3841.70640

**Published:** 2025-11-15

**Authors:** Deandrae Smith, Melissa Gonzalez Zuleta

**Affiliations:** ^1^ Department of Food Science Purdue University West Lafayette Indiana USA; ^2^ Department of Food Science and Technology Zamorano University Tegucigalpa Honduras

**Keywords:** cellulase, enzymatic hydrolysis, food industry wastewater, lipase, microwave processing, resource recovery

## Abstract

Meat and vegetable processing wastewaters are challenging to treat due to high concentrations of recalcitrant organic compounds—such as fats, oils, and cellulose—that resist conventional degradation. This study systematically evaluated the use of 915 MHz microwave (MW) processing, focusing on the effects of specific energy flux (SEF, in J/kg·s), total solids (TS) concentration, and flow rate on enzymatic activity and physicochemical properties. In vegetable processing wastewater, optimized MW conditions (SEF = 3,244.21 J/kg·s, TS = 2%) maximized cellulase activity (50.09 U/mL) and glucose release (0.1551 mg/mL), whereas higher TS (4%) reduced treatment efficacy. In meat processing wastewater, increased SEF (up to 2,775.51 J/kg·s) enhanced lipase activity (29.88 U/mL, 0.45 mg/mL), reduced viscosity and density, and altered the organic acid profile by increasing propionic acid and decreasing acetic acid. Statistical analysis confirmed SEF as the primary driver of biochemical and thermal changes (*R* ^2^ > 0.89). These results demonstrate that optimized 915 MHz MW processing is a scalable, sustainable, and regulatory‐compliant technology for food industry wastewater management. The approach improves enzymatic hydrolysis, biodegradability, and resource recovery, while reducing environmental impact and operational costs. Further research is recommended to validate these findings under full‐scale industrial conditions.

## 1 Introduction

Food processing is a cornerstone of the global food supply, but it is also a major consumer of freshwater and a significant generator of high‐strength wastewater. In the United States, food processing facilities are estimated to produce tens of billions of gallons of wastewater annually, contributing substantially to the overall industrial wastewater load (U.S. EPA 2023). Fruit and vegetable processing alone requires 1,000–2,000 L of water per ton of product and generates 5–10 m^3^ of effluent per ton (Sharma et al. 2016; Raffo and Paoletti 2022). These wastewaters are characterized by high concentrations of organic matter and suspended solids, presenting substantial challenges for sustainable management. A key distinction between vegetable and meat processing wastewaters lies in their principal organic constituents: cellulose predominates in vegetable effluents, accounting for up to 30% of chemical oxygen demand (COD), while fats, oils, and grease (FOG) are prevalent in meat effluents (Mishra et al. 2024; Zuppolini et al. 2022; Chipasa and Mędrzycka 2006; Bustillo‐Lecompte and Mehrvar 2017). Both cellulose and lipids are resistant to conventional biological degradation, with cellulose's crystalline structure and lipids’ tendency to form stable emulsions further complicating treatment (Bustillo‐Lecompte and Mehrvar 2017; Mishra et al. 2024).

Traditional treatment methods—such as filtration, dissolved air flotation (DAF), and chemical oxidation—are widely implemented but often face limitations, including high operational costs, slow reaction rates, and the generation of secondary pollutants (Gao et al. 2022; Hummel et al. 2025). Chemical oxidation, especially with hydrogen peroxide (H_2_O_2_), is used in some meat wastewater treatment applications due to its efficacy in degrading lipid‐based contaminants and its environmentally benign byproducts (Bustillo‐Lecompte and Mehrvar 2017; U.S. EPA 2023). However, the use of H_2_O_2_ is not universal and is often limited by cost and operational considerations. For cellulose‐rich vegetable effluents, H_2_O_2_ is generally less effective unless combined with catalysts or extreme conditions (Mishra et al. 2024; Zuppolini et al. 2022).

Vegetable and meat processing wastewaters present significant challenges due to the resistance of cellulose and lipid fractions to conventional degradation, highlighting the need for innovative and sustainable technologies that can efficiently target these compounds while minimizing environmental impact and operational costs (Smith and Atungulu 2018; Remya and Lin 2010). Microwave (MW) processing has emerged as a promising alternative, offering rapid and uniform heating via dipolar rotation and ionic conduction. Notably, the 915 MHz MW frequency provides deeper penetration and more homogeneous heating than the standard 2,450 MHz systems, making it particularly effective for the dense, heterogeneous matrices typical of food industry wastewaters (Smith and Atungulu 2018; Vialkova et al. 2021). Studies demonstrate that MW treatment at 915 MHz disrupts cellulose crystallinity, enhances enzymatic accessibility, and promotes lipid ester bond cleavage, thereby improving biodegradability and treatment efficiency (Remya and Lin 2010; Gupta and Singh 2020).

This approach is distinctive for its ability to deliver deep, uniform heating across large volumes, which is especially advantageous in industrial‐scale applications. By specifically targeting resistant organic compounds, microwave processing accelerates both chemical and physical breakdown, leading to more thorough solubilization of organic matter and enhanced bioavailability for subsequent biological treatment or anaerobic digestion. As a result, processing times are reduced, biogas yields are increased, and resource recovery is improved. Additionally, this technology supports sustainability by lowering energy consumption and enabling advanced process modeling and real‐time monitoring for optimized performance.

Despite these clear advantages, the full potential of 915 MHz microwave (MW) processing for the simultaneous degradation of cellulose and lipids in food processing wastewaters remains underexplored. Current research has demonstrated the technology's effectiveness in laboratory settings, but transitioning to industrial‐scale applications requires precise control and optimization of key parameters—such as specific energy flux (SEF), total solids concentration, and volumetric flow rate. These factors critically influence treatment efficiency, energy consumption, and process reliability, yet their interactions and optimal ranges for real‐world, heterogeneous waste streams are not well understood.

Failure to systematically optimize these variables could result in inconsistent pollutant removal, increased energy costs, or even reduced effectiveness of downstream biological treatments. Furthermore, as regulatory standards for wastewater quality and resource recovery become more stringent, there is an urgent need for robust, scalable solutions that can reliably meet these demands while minimizing environmental impact.

This study is among the first to systematically investigate 915 MHz MW processing for the efficient cleavage of cellulose and lipids in both vegetable and meat processing wastewaters. By methodically evaluating and optimizing MW parameters, this research aims to bridge the gap between laboratory findings and industrial implementation. The objectives are to enhance enzymatic activity, improve physicochemical conditions for downstream treatment, and ultimately advance sustainable wastewater management practices in the food industry. Through this work, we seek to support regulatory compliance, maximize resource recovery, and promote long‐term environmental stewardship—delivering practical, science‐driven solutions for the challenges facing modern food processing operations.

The objectives of this study are to systematically determine the optimal microwave (MW) processing parameters—specifically, SEF, total solids (TS) concentration, and flow rate—that maximize the degradation of cellulose and lipids in fruit, vegetable, and meat processing wastewaters while minimizing energy consumption. Additionally, the research aims to quantitatively evaluate changes in endogenous cellulase and lipase activities following MW exposure using established enzymatic assays. To assess the efficacy of MW treatment, the production of key degradation products—including glucose, acetic, butyric, formic, and propionic acids—will be measured, providing insight into the extent of hydrolysis and improvements in overall biodegradability. Finally, the study will characterize alterations in wastewater physicochemical properties such as temperature, viscosity, pH, and density, in order to elucidate process dynamics and potential impacts on microbial activity during downstream treatment.

By advancing MW‐based strategies for wastewater treatment, this research aims to reduce environmental impact, lower operational costs, and promote sustainable practices within the food processing industry, representing a novel application of 915 MHz MW technology for simultaneous cellulose and lipid degradation.

## 2 Materials and Methods

### 2.1 Sample Collection and Storage

Meat wastewater (4% total solids, TS) was collected from a commercial food processing facility (Independence Solutions, South Carolina, USA). Vegetable wastewater was sourced from two processors: Producer 1 (Independence Solutions, South Carolina, 2% TS) and Producer 2 (Mainland Solutions, North Carolina, 4% TS). All samples were transported in 1,250‐L intermediate bulk containers at 10°C and processed within 48 h to minimize microbial and enzymatic changes.

### 2.2 Meat Wastewater Pretreatment

Prior to microwave (MW) treatment, hydrogen peroxide (H_2_O_2_, 1.5% v/v) was added to meat wastewater at 10 mL/L and stirred for 15 min at 150 rpm using a standard industrial drum mixer in a 55‐gallon barrel. The mixer employed an impeller with an estimated blade radius of approximately 10 to 15 cm. The mixing speed is reported in rpm, and given the large impeller size, this corresponds to an estimated relative centrifugal force in the range of approximately 11,000 × g to 17,000 × g. MW treatment was performed immediately after pretreatment. H_2_O_2_ was not used for vegetable wastewater, as its efficacy for cellulose degradation is limited without catalysts or extreme conditions (Bustillo‐Lecompte and Mehrvar 2017).

### 2.3 Microwave (MW) Treatment

**FIGURE 1:**
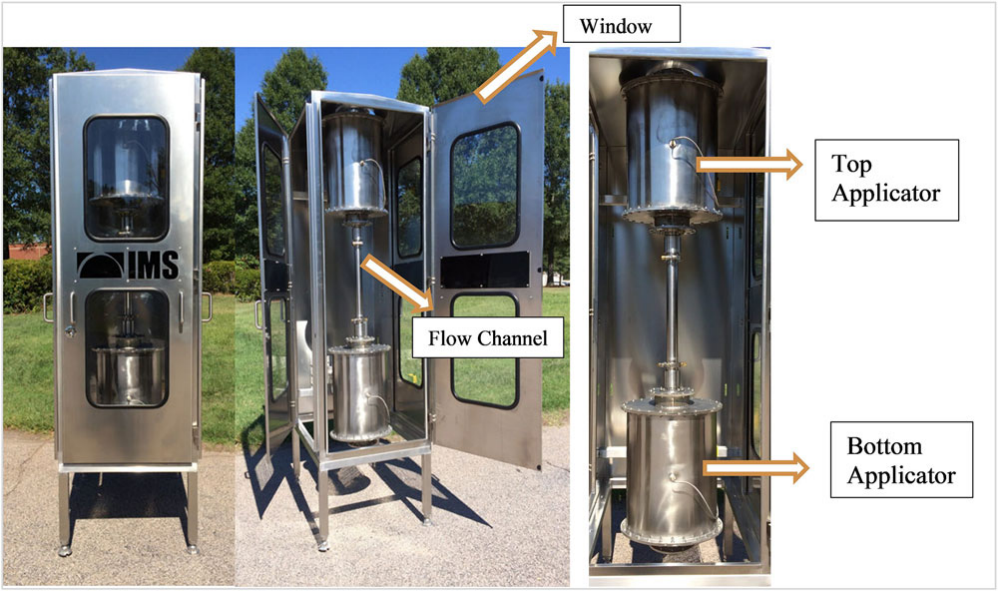
Front, side, and internal views of the 915 MHz liquid heating microwave system.

Continuous‐flow MW treatment was conducted using a 915 MHz liquid heating system (Microwave Techniques, LLC, Nashua, NH, USA) equipped with a vertical stainless‐steel chamber and dual applicators for uniform heating (see Figure 1 for schematic). Real‐time temperature monitoring was performed at the inlet, midpoint, and outlet using fiber optic sensors to avoid electromagnetic interference. MW power, SEF, TS concentration, and flow rate were systematically adjusted to achieve target temperatures, as detailed in Table 1. For lipid‐rich wastewater, the target temperature was 70–90°C to disrupt lipid crystallinity, enhance emulsification, and support lipase activity. For cellulose‐rich wastewater, treatments were performed at 40–50°C to optimize cellulase activity and at 70–90°C to promote thermal hydrolysis. All treatments were conducted in triplicate (biological replicates), and untreated samples served as negative controls Table 2a, 2b.

**TABLE 1:** Experimental parameters for microwave treatment: power, total solids, flow rate, and specific energy flux.

Wastewater sample	Power (kW)	Total solids (%)	Volumetric flow rate (m^3^/s)	SEF (J/kg.s)
Vegetable	47	2	1.89 × 10^4^	3,489.85
	48			3,244.21
	49			3,417.28
	41	4	1.74 × 10^4^	3,105.57
	42			3,186.17
	43			3,216.52
Meat	38	2	1.24 × 10⁻^2^	2,400.98
	39			2,459.53
	40			2,775.51

**TABLE 2a:** Summary of the statistical fit for key response variables for the vegetable wastewater study.

Wastewater sample	Response	Source	*p*‐value	*R*‐square	*R*‐square Adj.	Root mean square error
Vegetable	Temperature change (°C)	SEF (J/kg.s)	0.00001	0.99610	0.99566	2.46197
		Total solids (%)	0.00001			
		Total solids (%) *SEF (J/kg.s)	0.00001			
	Density (g/mL)	Total solids (%)	0.00001	0.89347	0.88786	0.00208
	pH			0.89241	0.88675	0.06220
	Cellulase activity (U/mL)			0.94400	0.93654	4.60277
		SEF (J/kg.s)	0.00001			
	Glucose (mg/mL)		0.00061	0.66847	0.62427	0.04636
		Total solids (%)	0.00061			
	Butyric acid (mg/mL)		0.00001	0.83900	0.81753	0.135407
		Total solids (%) *SEF (J/kg.s)	0.00961			
	Formic acid (mg/mL)	Total solids (%) *SEF (J/kg.s)	0.00743	0.40059	0.36904	0.05536
		SEF (J/kg.s)	0.04769			
	Propionic acid (mg/mL)	Total solids (%)	0.01171	0.29040	0.25305	0.05534

**TABLE 2b:** Summary of the statistical fit for key response variables for the meat wastewater study.

Wastewater sample	Response	Source	*p*‐value	*R*‐Square	*R*‐square Adj.	Root mean square error
Meat	Density (g/mL)	SEF (J/kg.s)	< 0.0001	0.96714	0.95482	0.00336
	Viscosity (Pa·s)	SEF (J/kg.s)	0.0206	0.68628	0.56863	0.00408
	pH	SEF (J/kg.s)	0.0135	0.71887	0.61347	0.01414
	Acetic acid (mg/mL)	SEF (J/kg.s)	0.0103	0.73779	0.63496	0.04301
	Propionic acid (mg/mL)	SEF (J/kg.s)	< 0.0001	0.97478	0.96532	0.04528
	Butyric acid (mg/mL)	SEF (J/kg.s)	0.0089	0.78123	0.69234	0.03312
	Formic acid (mg/mL)	SEF (J/kg.s)	0.0121	0.75614	0.65841	0.02489
	Lipase concentration (mg/mL)	SEF (J/kg.s)	0.0024	0.89256	0.84743	0.04963
	Enzymatic activity (UI/mL)	SEF (J/kg.s)	0.0011	0.93945	0.91327	2.37851

### 2.4 Physicochemical Analyses

#### 2.4.1 pH

**pH** was measured at 25°C using a calibrated Mettler Toledo SD23 meter (buffers pH 4, 7, 10).

#### 2.4.2 Density

Density was determined gravimetrically as *ρ* = *m/V* using 25 mL samples and a calibrated analytical balance. Heat transfer (Q, J) was calculated as *Q* = *P* × *t*, where *P* is power input (W) and t is exposure time (s). SEF (J·kg^−1^·s^−^) was calculated as SEF = *Q/ṁ*, where ṁ is mass flow rate (kg/s) (Smith and Atungulu 2018).

### 2.5 Enzyme Activity Assays

Endogenous enzyme activities were measured to assess the microbial enzymatic response to MW treatment. No exogenous enzymes were added. All assays included sample blanks (wastewater without substrate) to correct for background absorbance and color interference.

#### 2.5.1 Lipase Activity

Lipase activity in meat wastewater was quantified using the p‐nitrophenyl palmitate (pNPP) assay. Samples (5 mL) were centrifuged (10,000 rpm, 10 min); 3 mL supernatant was mixed with 2 mL 1% pNPP (in 0.1 M phosphate buffer, pH 7.2), incubated at 37°C for 70 min, and stopped with 1 mL sodium carbonate. Absorbance was measured at 410 nm (Cary 8454, Agilent), with blank correction. Activity (U/mL) was calculated from a p‐nitrophenol standard curve (0.2–1.2 mM; *R* ^2^ = 0.9994) as described by Michalak et al. (2017).

#### 2.5.2 Cellulase Activity

Cellulase activity in vegetable wastewater was measured using the 3,5‐dinitrosalicylic acid (DNS) method. Samples (5 mL) were centrifuged; 3 mL supernatant was mixed with 2 mL 1% carboxymethyl cellulose (CMC) in 0.1 M acetate buffer (pH 5.0) and incubated at 25°C for 70 min. After the reaction, 1 mL of mixture plus 1 mL of DNS reagent was heated at 90°C for 120 min. Absorbance was read at 540 nm. Activity (U/mL) was calculated from a glucose standard curve (0.2–1.2 mg/mL; *R* ^2^ = 0.9994) (Miller 1959).

### 2.6 Glucose and Organic Acid Analysis

Glucose, formic, acetic, butyric, and propionic acids were quantified by high‐performance liquid chromatography (HPLC; Thermo Scientific Ultimate 3000) using an Aminex HPX‐87H column with 0.005 M H_2_SO_4_ as the mobile phase (0.60 mL/min, 35 min, 90°C). Glucose was detected by refractive index, and organic acids at 210 nm. Standard solutions were serially diluted, acidified, and centrifuged before triplicate analysis. Concentrations were determined using linear calibration curves (Gupta and Singh 2020).

### 2.7 Statistical Analysis

Data were analyzed using SAS software (SAS Institute, Cary, NC, USA). Multiple regression was used to assess relationships between process variables (e.g., SEF, TS) and outcomes (e.g., temperature, glucose concentration). Statistical significance was set at *p* < 0.05. Results are reported as mean ± standard deviation.

## 3 Results and Discussion

### 3.1 Statistical Modeling of Process Variables

In vegetable wastewater, SEF and TS strongly influenced key responses. Temperature change showed an excellent fit (*R* ^2^ = 0.9961), driven by SEF, TS, and their interaction. TS significantly affected density (*R* ^2^ = 0.8935). pH and cellulase activity models were strong (*R* ^2^ > 0.89). Glucose was moderately predicted (*R* ^2^ = 0.67). Butyric acid responded significantly to SEF and TS interaction, while formic and propionic acids showed moderate to weak effects, reflecting complex fermentation.

In meat wastewater, SEF was the main driver, with strong effects on density (*R* ^2^ = 0.9671), pH, viscosity, and organic acids (*R* ^2^ up to 0.97). SEF also markedly increased lipase concentration and activity (*R* ^2^ > 0.89), confirming microwave treatment's role in enhancing biochemical processes.

### 3.2 Physicochemical Changes Induced by Microwave (MW) Treatment

#### 3.2.1 Temperature Profiles

MW treatment resulted in significant temperature increases in both meat and vegetable wastewaters, strongly correlated with SEF and TS concentration. In meat wastewater, the highest SEF (2775.51 J/kg·s) raised the temperature by 56.4°C, reaching 105.7°C, while lower SEF values induced smaller increases (Table 3). For vegetable wastewater, temperature increases were also dependent on both TS and SEF, with a maximum rise of 63.7°C observed at 4% TS and 41 kW (SEF = 3105.57 J/kg·s) (Table 3). These findings confirm that MW energy input and solids content are primary determinants of heating efficiency, consistent with prior MW‐assisted wastewater treatment studies (Zhu and Blackborow 2023).

**TABLE 3:** Influence of specific energy flux, total solids, and initial temperature on wastewater final temperature and ΔT.

Wastewater sample	SEF (J/kg.s)	Total solids (%)	Initial temperature (°C)	Final temperature (°C)	Temperature change (°C)
Vegetable	3,489.85	2	9.61	45.91	36.30
	3,244.21			43.08	33.47
	3,417.28			41.42	31.81
	3,105.57	4		73.33	63.72
	3,186.17			81.11	71.50
	3,216.52			85.00	75.39
Meat	2,400.98	2	49.3	55.60	6.30
	2,459.53			57.80	8.50
	2,775.51			105.70	56.40

#### 3.2.2 Density and Viscosity Changes

MW processing reduced density and viscosity in meat wastewater, thereby improving flow characteristics. Density decreased from 1.07 g/mL (control) to 1.029 g/mL at the highest SEF, while viscosity declined from 0.03 Pa·s to 0.02 Pa·s at 2775.51 J/kg·s (Table 4). In vegetable wastewater, density was mainly influenced by TS concentration; higher TS led to increased density, with MW treatment exerting minimal effects within TS groups (Table 4). These reductions likely result from enhanced molecular motion and breakdown of complex organic matrices (Smith and Jones 2023; Brown 2023; Sharma 2023).

**TABLE 4:** Effects of specific energy flux and total solids on wastewater viscosity, density, and pH.

Wastewater sample	SEF (J/kg.s)	Total solids (%)	pH	Density (g/mL)	Viscosity (Pa·s)
Vegetable	Control	2	7.24 ± 0.02^A^	1.02 ± 0.00^B^	N/A
	3,489.85		7.10 ± 0.06^B^	1.02 ± 0.00^B^	
	3,244.21		7.01 ± 0.06^C^	1.01 ± 0.00^B^	
	3,417.28		7.03 ± 0.02^C^	1.02 ± 0.00^B^	
	Control	4	6.84 ± 0.01^D^	1.03 ± 0.00^A^	
	3,186.17		6.80 ± 0.00^D^	1.03 ± 0.00^A^	
	3,216.52		6.78 ± 0.03^D^	1.03 ± 0.00^A^	
Meat	Control	2	5.29 ± 0.02^B^	1.07 ± 0.019^A^	0.03 ± 0.0024^A^
	2,400.98		5.32 ± 0.02A^B^	1.035 ± 0.003^B^	0.033 ± 0.003^A^
	2,459.53		5.33 ± 0.01A^B^	1.030 ± 0.002^B^	0.027 ± 0.003^B^
	2,775.51		5.34 ± 0.01^A^	1.029 ± 0.002^B^	0.02 ± 0.002^C^

*Note*: Letters denote significant differences, indicating statistically distinct values ​within the column at *p* ≤ 0.05.

#### 3.2.3 pH Modulation

MW treatment caused a slight increase in pH in meat wastewater, from 5.29 to 5.34, while vegetable wastewater exhibited a pH decrease, from 7.24 to 7.01 to 7.10 at 2% TS and even lower at higher TS levels (Table 4). The pH decline in vegetable samples is attributed to increased organic acid release, whereas the pH rise in meat wastewater likely reflects enhanced ionization and acid‐base equilibria induced by MW exposure (McNamara et al. 2015; Helmenstine 2023).

#### 3.2.4 Cellulase Activity in Vegetable Wastewater

Cellulase activity markedly increased under optimal MW power at 2% TS, reaching 50.09 U/mL (48 kW, SEF = 3244.21 J/kg·s), compared to 6.44 U/mL in controls (Table 5). However, at 4% TS, activity was notably lower, indicating excessive solids may hinder enzyme accessibility or MW penetration. These results emphasize the importance of optimizing both SEF and TS to maximize enzymatic hydrolysis efficiency (Ha et al. 2011; Adney and Baker 1996).

**TABLE 5:** Influence of energy input and solids content on enzymatic activity and glucose release in vegetable wastewater.

SEF (J/kg.s)	Total solids (%)	Cellulase activity (U/mL)	Glucose (mg/mL)
Control	2	6.44 ± 0.90^E^	0.01 ± 0.02^B^
3,489.85		42.36 ± 1.89^B^	0.14 ± 0.00^A^
3,244.21		50.09 ± 1.43^A^	0.16 ± 0.00^A^
3,417.28		38.07 ± 2.03^C^	0.14 ± 0.01^A^
Control	4	06.06 ± 0.50^E^	0.01 ± 0.01^B^
3,186.17		07.15 ± 0.65^E^	0.00 ± 0.00^C^
3,216.52		13.64 ± 0.97^D^	0.06 ± 0.11^B^

*Note*: Letters denote significant differences, indicating statistically distinct values ​within the column at *p* ≤ 0.05.

### 3.3 Carbohydrate and Organic Acid Transformations

#### 3.3.1 Glucose Release

MW treatment substantially enhanced glucose release in vegetable wastewater at 2% TS, increasing from 0.01 mg/mL (control) to 0.16 mg/mL at 48 kW (Table 5). At 4% TS, glucose yields were reduced, consistent with decreased cellulase activity under higher solids content. These results confirm MW processing facilitates carbohydrate breakdown under optimal SEF and TS (Azuma et al. 1984; Zhu et al. 2005; Hu and Wen 2008).

### 3.4 Enhancement of Enzymatic Hydrolysis

#### 3.4.1 Lipase Activity in Meat Wastewater

MW processing significantly elevated endogenous lipase concentration and activity, with the highest SEF yielding 0.45 mg/mL and 29.88 UI/mL, respectively, compared to 0.09 mg/mL and 4.22 UI/mL in controls (Table 6). This enhancement suggests MW‐induced thermal and non‐thermal effects promote lipid hydrolysis, supporting previous findings on MW‐facilitated enzyme activation (Brock et al. 2023; Singh and Mukhopadhyay 2023; Nelson and Cox 2023; Voet and Voet 2023).

**TABLE 6:** Effect of specific energy flux on the viscosity of meat wastewater.

SEF (J/kg·s)	Lipase (mg/mL)	Enzymatic activity (UI/mL)
Control	0.09 ± 0.02^C^	4.22 ± 1.01^C^
2,400.98	0.19 ± 0.01^B^	11.37 ± 0.85^B^
2,459.53	0.29 ± 0.02^A^	18.73 ± 1.52^A^
2,775.51	0.45 ± 0.03^A^	29.88 ± 2.14^A^

*Note*: Letters denote significant differences, indicating statistically distinct values ​within the column at *p* ≤ 0.05.

#### 3.4.2 Organic Acid Profiles

In meat wastewater, acetic acid concentrations decreased with increasing SEF, from 1.29 mg/mL (control) to 1.18 mg/mL at the highest SEF, while propionic acid increased from 4.13 to 4.67 mg/mL (Table 8). Butyric and formic acids remained relatively stable. In vegetable wastewater, butyric acid rose with MW power at 2% TS but decreased at 4% TS (Table 7). Formic acid was consistently higher at 2% TS, and propionic acid production was enhanced by MW treatment at moderate power levels. These shifts reflect MW‐driven modulation of microbial metabolic pathways, influencing acidogenesis and resource recovery potential (Cao et al. 2021; Salvachua et al. 2021; Detman et al. 2019; Chukwudebelu and Agunwamba 2017; Fang and Chen 2021; Franco and Taraborrelli 2021; Yap et al. 2024; Ricci‐Jürgensen and Confalonieri 2016; Akpor et al. 2014).

**TABLE 7:** Influence of energy flux and total solids content on short‐chain fatty acids in vegetable wastewater.

SEF (J/kg.s)	Total solids (%)	Volumetric flow rate (m^3^/s)	Butyric acid (mg/mL)	Formic acid (mg/mL)	Propionic acid (mg/mL)
Control	2	1.89 × 10^4^	0.36 ± 0.11^C^	0.08 ± 0.02^A^	0.014 ± 0.02^B^
3,489.85			0.65 ± 0.09^B^	0.17 ± 0.06^A^	0.00 ± 0.00^B^
3,244.21			0.64 ± 0.05^B^	0.15 ± 0.04^A^	0.00 ± 0.00^B^
3,417.28			0.60 ± 0.09^B^	0.13 ± 0.01^A^	0.00 ± 0.00^B^
Control	4	1.74 × 10^4^	0.83 ± 0.33^B^	0.01 ± 0.01^B^	0.04 ± 0.03^A^
3,186.17			1.16 ± 0.19^A^	0.01 ± 0.09^B^	0.12 ± 0.10^A^
3,216.52			1.09 ± 0.25^A^	0.05 ± 0.07^B^	0.06 ± 0.10^A^

*Note*: Letters denote significant differences, indicating statistically distinct values ​within the column at *p* ≤ 0.05.

**TABLE 8:** Influence of energy input on short‐chain fatty acid profiles in meat wastewater.

SEF (J/kg·s)	Acetic acid (mg/mL)	Propionic acid (mg/mL)	Butyric acid (mg/mL)	Formic acid (mg/mL)
Control	1.29 ± 0.02^A^	4.13 ± 0.05^C^	2.03 ± 0.02^A^	0.44 ± 0.01^A^
2,400.98	1.14 ± 0.01^B^	4.25 ± 0.05^B^	2.05 ± 0.01^A^	0.43 ± 0.01^A^
2,459.53	1.16 ± 0.01^B^	4.61 ± 0.03^A^	2.08 ± 0.02^A^	0.41 ± 0.01^A^
2,775.51	1.18 ± 0.02^B^	4.67 ± 0.03^A^	2.11 ± 0.03^A^	0.45 ± 0.01^A^

*Note*: Letters denote significant differences, indicating statistically distinct values ​within the column at *p* ≤ 0.05.

### 3.5 Relevance to Food Science and Sustainable Processing

This study demonstrates that MW treatment, optimized for SEF and TS, significantly enhances degradation of recalcitrant organics and enzymatic hydrolysis in meat and vegetable processing wastewaters. These improvements in physicochemical properties and bioconversion efficiency have direct implications for sustainable wastewater management, resource recovery, and environmental compliance in the food industry. The findings align with current trends in food science, emphasizing innovative, scalable, and environmentally responsible processing technologies (Gupta and Singh 2020).

## 4 Conclusions

This study demonstrates that optimized 915 MHz microwave (MW) processing offers a promising, energy‐efficient approach for treating meat and vegetable processing wastewaters, which are characterized by high concentrations of recalcitrant organic compounds that often challenge conventional wastewater treatment methods. Through systematic investigation, SEF and TS concentration were identified as critical parameters influencing MW treatment efficacy. Experimental results showed that appropriate MW conditions enhanced enzymatic activity—notably cellulase in vegetable wastewaters and lipase in meat wastewaters—and improved the physicochemical properties of the effluents, thereby increasing biodegradability and processability.

In meat processing wastewaters, higher SEF values led to increased lipase activity and lipid hydrolysis, as evidenced by elevated concentrations of propionic acid and improved flow properties (reduced viscosity and density), with minor pH increases indicative of chemical transformations. In vegetable processing wastewaters, optimal MW treatment maximized cellulase activity and glucose release, although higher TS levels were found to reduce treatment efficacy. MW exposure also altered the organic acid profile, with significant increases in butyric acid and variable effects on formic and propionic acids depending on treatment conditions.

Statistical analysis confirmed that SEF was the primary driver of biochemical and thermal changes, with heating efficiency strongly dependent on both SEF and TS (*R* ^2^ > 0.89). The findings bridge the gap between laboratory‐scale research and industrial‐scale application, addressing the need for robust, sustainable, and regulatory‐compliant wastewater management solutions in the food industry.

By improving enzymatic activity and optimizing downstream process conditions, this research advances MW‐based strategies for resource recovery and environmental stewardship. These results highlight the potential of 915 MHz MW technology to reduce environmental impact, lower operational costs, and support sustainable practices in food processing.

## Author Contributions

**Deandrae Smith**: conceptualization, funding acquisition, methodology, writing – review and editing, project administration, supervision, resources. **Melissa Gonzalez Zuleta**: formal analysis, writing – original draft, investigation, data curation.

## Conflicts of Interest

The authors declare no conflicts of interest.
